# Unusual presentation of colonic angiodysplasia with recurrent ischemia and venous thrombosis

**DOI:** 10.1093/jscr/rjaf1068

**Published:** 2026-01-15

**Authors:** Ahmed Alanzi, Bano Alsaleh, Dawood Alatefi, Heet Sheth, Khaled M AlAani, Ajaz Wani, Fouad Aladel

**Affiliations:** Anaesthesia and Pain Management Department, King Hamad University Hospital, Building 2345, Road 2835, Busaiteen 228, Muharraq, Kingdom of Bahrain; Radiology Department, King Hamad University Hospital, Building 2345, Road 2835, Busaiteen 228, Muharraq, Kingdom of Bahrain; Yemeni-Syrian Medical Center, Ja'ar, Abyan Governorate, Yemen; Royal College of Surgeons in Ireland – Bahrain, PO Box 15503, Building 2441, Road 2835, Busaiteen 228, Muharraq, Kingdom of Bahrain; Faculty of Medicine and Medical sciences, Arabian Gulf University, Building 293, Road 2904, Block 329, PO Box 26671, Manama, Kingdom of Bahrain; General Surgery Department, King Hamad University Hospital, Building 2345, Road 2835, Busaiteen 228, Muharraq, Kingdom of Bahrain; Radiology Department, King Fahad Specialist Hospital, Ammar Bin Thabit St, Al Merikbat neighborhood, Dammam, 32253, Saudi Arabia

**Keywords:** colonic angiodysplasia, ischemic colitis, CT angiography, venous thrombosis, inferior vena cava thrombosis, recurrent ischemia

## Abstract

Colonic angiodysplasia is a common vascular malformation of the gastrointestinal tract, typically presenting with lower gastrointestinal bleeding. Ischemic complications are rare, and their association with systemic venous thrombosis has not been reported. We describe a 39-year-old man with end-stage renal disease, diabetes insipidus, and recurrent venous thromboses who presented with abdominal pain and sepsis. Computed tomography revealed colonic angiodysplasia, ischemic bowel, and a non-occlusive inferior vena cava thrombus. He underwent transverse and left hemicolectomy with stoma formation, followed by a second-look laparotomy and ileocolic anastomosis; an IVC filter was inserted. Three months later, he re-presented with peritonism and new iliac and femoral vein thrombi. Laparotomy confirmed diffuse ischemia of terminal ileum with venous engorgement but preserved arterial inflow. Histopathology demonstrated mucosal ischemia with dilated submucosal vessels. This case highlights a novel pathophysiological mechanism in which venous hypertension from systemic thrombosis exacerbates angiodysplasia, leading to recurrent ischemia despite surgical resection and filtration.

## Introduction

Colonic angiodysplasia is the most common vascular malformation of the gastrointestinal tract and a frequent cause of lower gastrointestinal bleeding, particularly in older adults [1]. Lesions are typically located in the cecum and ascending colon where wall tension favors vascular ectasia [2]. Patients usually present with occult or overt bleeding, while ischemic manifestations are rarely described [3]. Risk factors such as chronic kidney disease, aortic stenosis (Heyde’s syndrome), and acquired von Willebrand disease contribute to vascular fragility and impaired hemostasis [4–6]. Although venous congestion is implicated in the pathogenesis of angiodysplasia [7], ischemia is generally attributed to arterial hypoperfusion or systemic circulatory compromise. Venous causes of colitis are less common and usually involve inferior mesenteric vein thrombosis [8]. We present a unique case of colonic angiodysplasia complicated by inferior vena cava (IVC) and iliac vein thromboses, leading to recurrent ischemia despite colectomy and IVC filtration, underscoring the importance of recognizing venous-hypertension mechanisms.

## Case presentation

A 39-year-old man with a history of end-stage renal disease (ESRD) on maintenance hemodialysis, diabetes insipidus, and recurrent venous thromboembolism presented to the emergency department with acute abdominal pain, fever, and lethargy. He was a lifelong non-smoker, denied alcohol or illicit drug use, and had no family history of thrombophilia, inflammatory bowel disease, or gastrointestinal malignancy. He had been on long-term anticoagulation with apixaban for two years due to prior venous thromboses. On arrival, he was hypotensive (blood pressure 90/65 mmHg) and tachycardic (pulse 122 bpm), with mild tachypnea. Laboratory tests revealed leukocytosis (WBC 15.2 × 10^9^/L), elevated C-reactive protein (CRP 215 mg/L), metabolic acidosis (lactate 5.8 mmol/L), and impaired renal function consistent with his baseline ESRD.

**Figure 1:**
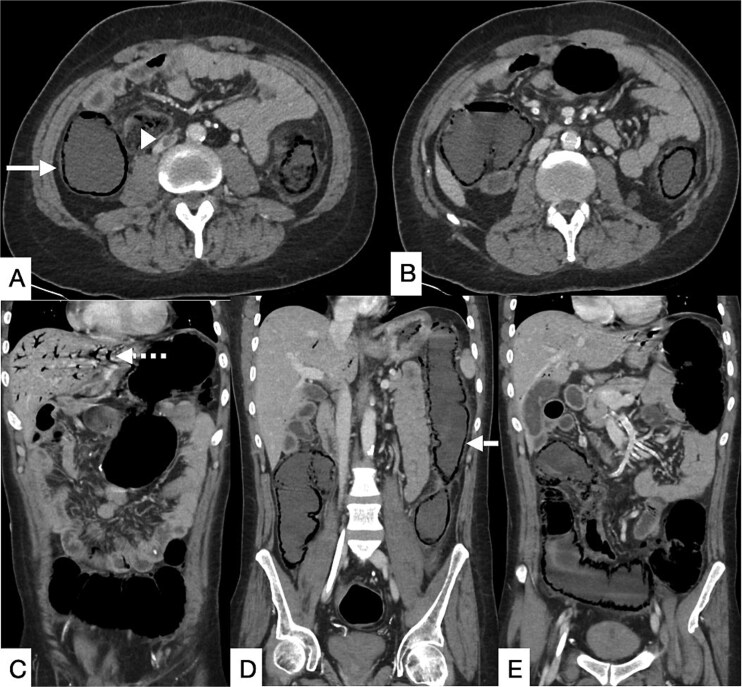
Contrast-enhanced CT of the abdomen and pelvis in axial (A, B) and coronal (C–E) planes showing a long segment of pneumatosis intestinalis involving the remaining descending colon, transverse colon, and proximal sigmoid colon, with suspicion of bowel perforation (bold arrow). Pneumobilia is present (dashed arrow). An eccentric intraluminal hypodensity within the IVC (arrowhead) is consistent with a non-occlusive thrombus, with a possible thrombus in the inferior mesenteric vein.

**Figure 2:**
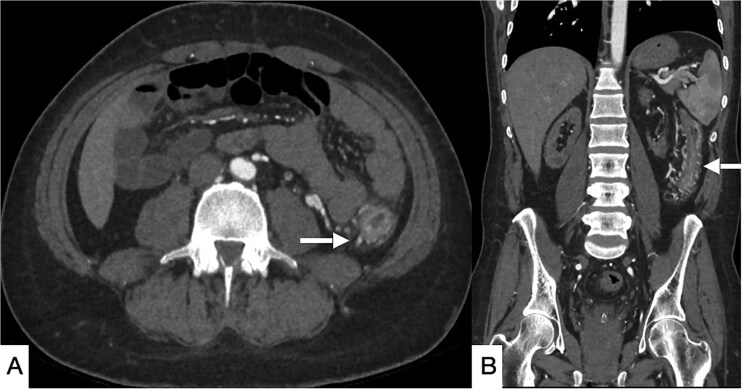
Selected CT angiogram of the abdomen and pelvis in axial (A) and coronal (B) planes showing mild diffuse thickening and mucosal hyper-enhancement of the descending colon, with prominent feeding mesenteric arteries and early draining vein dilation, consistent with angiodysplasia.

Contrast-enhanced computed tomography (CT) of the abdomen demonstrated long-segment ischemia extending from the transverse to descending colon, characterized by wall thickening, mucosal hypoenhancement, and pericolic fat stranding. A non-occlusive thrombus was noted in the IVC (Fig. 1). Review of prior CT angiography, performed several months earlier for gastrointestinal bleeding, had shown features of colonic angiodysplasia, including mucosal hyperenhancement, early venous filling, and serpiginous submucosal vessels (Fig. 2).

Given the findings, the patient underwent urgent laparotomy. Intraoperatively, the transverse and descending colon appeared dusky, thickened, and nonviable, while the small bowel and rectum remained viable. A transverse and left hemicolectomy with end colostomy was performed. The postoperative course was initially stable, and a planned second-look laparotomy 48 hours later confirmed viable remaining bowel. Ileocolic anastomosis was carried out at that stage. An IVC filter was inserted due to the patient’s recurrent thrombotic history and the concern for ongoing embolic phenomena.

Histopathological examination of the resected specimen revealed ischemic changes with dilated submucosal vessels, consistent with angiodysplasia.

**Figure 3:**
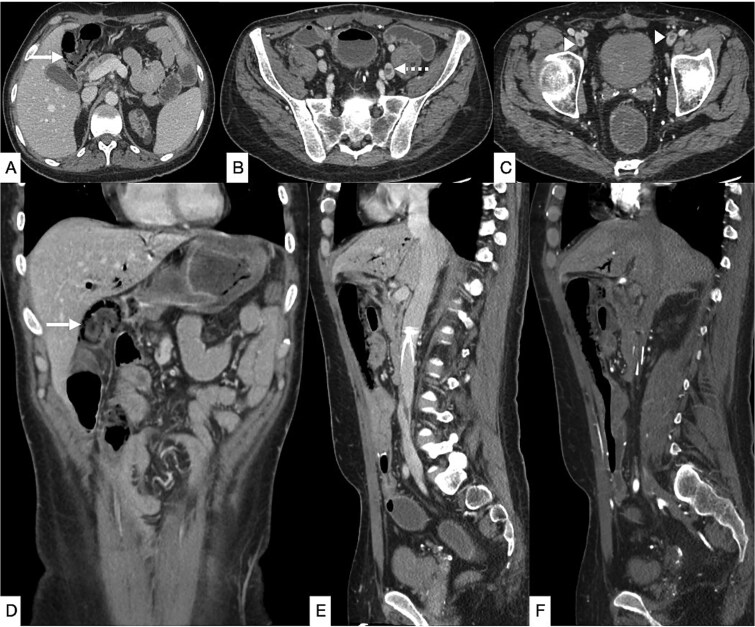
Contrast-enhanced CT of the abdomen and pelvis in axial (A–C), coronal (D), and sagittal (E, F) planes showing diffuse pneumatosis intestinalis just proximal to the previous anastomotic site (bold arrow), consistent with bowel ischemia. New thrombi are identified within the left common iliac vein (dashed arrow) and bilateral common femoral veins (arrowhead). Mild residual pneumobilia is present. An IVC filter is seen *in situ*.

Three months later, the patient re-presented with severe right iliac fossa pain radiating to the left abdomen, associated with fever and clinical peritonism. Laboratory evaluation again demonstrated metabolic acidosis, leukocytosis, and elevated inflammatory markers. CT of the abdomen demonstrated pneumatosis intestinalis in the terminal ileum adjacent to the ileocolic anastomosis, together with new thrombi in the left common iliac and bilateral femoral veins (Fig. 3).

An emergency exploratory laparotomy was performed. Approximately 25 cm of terminal ileum was diffusely ischemic, demonstrating venous engorgement with preserved arterial inflow. The affected segment was resected, and an end ileostomy was fashioned. The resected specimen showed patchy ischemia. Histopathology once again demonstrated mucosal ischemia with dilated, thin-walled angiodysplastic vessels.

The patient recovered uneventfully following the second operation. Hematology consultation recommended further hypercoagulable work-up, including testing for inherited thrombophilia, antiphospholipid syndrome, and myeloproliferative neoplasms. At the time of reporting, these investigations were ongoing and no definitive etiology for his recurrent venous thromboses had been identified.

## Discussion

Angiodysplasia usually manifests with gastrointestinal bleeding, most often in the right colon, and ischemic complications are distinctly uncommon [1–3]. Our patient’s course was atypical, as recurrent ischemia developed in the setting of systemic venous thromboses. This highlights the importance of considering alternative pathophysiological mechanisms beyond the classical bleeding presentation.

Several conditions predispose to angiodysplasia. Chronic kidney disease increases vascular fragility and impairs hemostasis, while aortic stenosis and acquired von Willebrand disease (Heyde’s syndrome) contribute through altered shear stress and loss of high-molecular-weight multimers [4–6]. Venous congestion is thought to play a role in the pathogenesis by causing intermittent obstruction of submucosal veins, leading to dilatation and vascular ectasia [7]. However, ischemia in angiodysplasia is generally attributed to arterial hypoperfusion or systemic shock rather than venous outflow obstruction.

Venous causes of colitis have been reported rarely, most often in association with inferior mesenteric vein (IMV) thrombosis [8]. These cases demonstrate localized colonic ischemia due to impaired venous drainage. In contrast, our case is unique because the ischemic changes were driven by systemic venous thromboses involving the IVC and iliac veins rather than mesenteric veins. This mechanism generated venous hypertension superimposed on the fragile angiodysplastic microvasculature, resulting in ischemia despite preserved arterial inflow. To our knowledge, this interplay has not been previously described in the literature.

Radiologically, CT angiography is considered the gold standard for diagnosing angiodysplasia, with sensitivity and specificity exceeding 90% [9]. Classic findings include mucosal hyperenhancement, early opacification of draining veins, and serpiginous submucosal vessels [10]. In our patient, these features were present on prior imaging, establishing the diagnosis before ischemic complications occurred. Follow-up imaging demonstrated pneumatosis intestinalis and new thrombi within the iliac and femoral veins, underscoring the need for radiologists to evaluate both arterial and venous systems when assessing ischemic bowel. Importantly, operative findings of venous engorgement with intact arterial inflow further supported a venous-hypertension–driven mechanism.

From a surgical standpoint, angiodysplasia-related ischemia is exceptionally rare, and intraoperative decision-making is complex. In this case, initial colectomy with staged anastomosis was successful, but recurrent ischemia developed despite IVC filtration, highlighting the limitations of surgical and interventional strategies when the underlying driver is systemic thrombosis. These findings emphasize the importance of early multidisciplinary involvement**,** including hematology for thrombophilia testing and optimization of anticoagulation.

The novelty of this case lies in demonstrating that systemic venous thromboses can precipitate recurrent ischemia in colonic angiodysplasia. Previous reports have described IMV thrombosis or arteriovenous malformations leading to localized venous hypertension [8, 11], but none have reported recurrent ischemia due to widespread venous thrombosis in this context. This case therefore expands the spectrum of clinical presentations and provides insight into the pathophysiological mechanisms linking systemic venous hypertension and colonic angiodysplasia.

## Conclusion

Colonic angiodysplasia usually presents with bleeding, but this case demonstrates that it can also lead to recurrent ischemia when compounded by systemic venous thromboses. The coexistence of venous outflow obstruction and fragile angiodysplastic vessels produced ischemia despite preserved arterial inflow, a mechanism not previously reported. Awareness of this presentation, careful radiological assessment of both venous and arterial systems, and early multidisciplinary management are essential to optimize outcomes.
